# Whey permeate powder is a suitable ingredient for ice cream

**DOI:** 10.3168/jdsc.2023-0382

**Published:** 2023-09-18

**Authors:** Derek Schweiger, Jack Myers, Stephanie Clark

**Affiliations:** 1 Department of Food Science and Human Nutrition, Iowa State University, Ames, IA 50011; 2 Department of Agricultural Economics and Agribusiness, University of Arkansas, Fayetteville, AR 72701

## Abstract

The objective of this research was to evaluate the suitability of whey permeate powder for ice cream. Three white mixes were formulated with equivalent total solids, fat, and carbohydrates, but different concentrations of lactose and added sugar. Vanilla ice creams contained either reduced lactose (RL, 3.8% lactose and 17% added sugar), standard lactose (SL, 5.8%; 15%), or high lactose (HL, 7.8%; 13%). Trained panelists evaluated 8 body and texture, and 6 flavor characteristics through 10 mo of storage. All ice creams maintained low mean scores (<4.0/15.0 cm) for crumbly, lacks freshness, nonfat dry milk flavor, and whey, and moderate mean scores (5.0–8.3/15.0 cm) for gummy, melt rate, melt viscosity, sweet, and vanilla flavor for 10 mo. In mo 1 and 10, consumers in Iowa (n = 94, n = 55) and in mo 4 and 6, consumers in Kansas (n = 44; n = 56) rated the acceptability of the ice creams. Overall acceptability, flavor, and texture acceptability for products did not significantly differ until mo 10, when HL mean scores decreased lower than SL ice cream mean scores. The lower scores are attributed to crumbly and sandy texture defects, noted by trained panelists, only for HL ice cream stored 10 mo. The research demonstrates that whey permeate powder can be used to produce ice creams of acceptable quality for up to 10 mo.

## Highlights

• Ice cream with whey permeate powder was as acceptable as ice cream without it for 7 months.
• High lactose concentration (7.8%) did not cause sandy defect in ice cream until month 10.
• Whey permeate powder is a suitable ingredient for ice cream applications.
• Substitution of whey protein concentrate (WPC80) with whey permeate powder could reduce production costsn.

## Graphical Abstract

**Figure:**
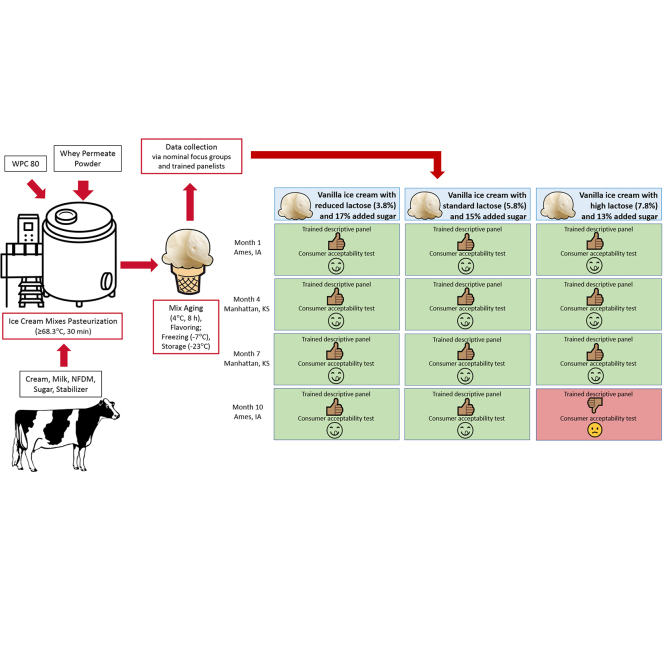
**Summary:** Whey permeate powder can be effectively used in ice cream that may undergo extended storage, even at lactose levels that were previously considered too high for ice cream.

Creating foods with lower added sugars and clean labels has become a priority for the industry. Highly soluble, with a mild dairy flavor, whey permeate powder (**WPP**), a co-product from the production of whey protein concentrate (**WPC**) and whey protein isolate (**WPI**), has been reported to be one of the most cost-competitive sources of dairy solids on the market (US Dairy Export Council, 2022). Furthermore, the utilization of co-products like WPP contributes to sustainability initiatives, as the dairy industry seeks to achieve net zero carbon emissions by 2050 (McComb, 2020).

Whereas the supply of lactose-rich ingredients has been growing because of the production of and demand for higher protein dairy ingredients (MPC, WPC, and WPI), the demand for high-lactose ingredients has not kept up. Though the clinical condition lactose intolerance is not common (~10% of the population), varying degrees of lactose maldigestion and malabsorption are common (~66% of the population) worldwide (Oak and Jha, 2019; Seoane et al., 2020; Jansson-Knodell et al., 2022). Additionally, some consumers wrongly associate symptoms of a variety of intestinal disorders with lactose intolerance, without undergoing testing, and therefore unnecessarily avoid dairy foods (Suchy et al., 2010). Furthermore, evidence suggests that even those medically diagnosed with lactose intolerance can ingest at least 12 g of lactose (equivalent to 1 cup of milk) at a single sitting without symptoms, and even more if consumed with meals and distributed throughout the day (Suchy et al., 2010).

Another limitation of WPP utilization is its high lactose content (approximately 82%). The complex chemistry of lactose limits its use in various applications, including ice cream. The hemiacetal structure enables the molecule to interchange between α- and β-anomers by mutarotation (Wong and Hartel, 2014), which is greatly influenced by temperature, concentration, pH, and other substances (Haase and Nickerson, 1966; Patel and Nickerson, 1970; Hartel and Shastry, 1991). While α-lactose is a soft, very fine, crystalline material that is not objectionable in foods, α-lactose monohydrate is a hard crystalline material with a gritty (or “sandy”) texture (Thomas et al., 2009), and is promoted during typical freeze-thaw cycles of frozen storage (Nickerson, 1962).

The surplus of lactose-rich ingredients necessitates research to increase its utilization. The objective of this research was to evaluate the suitability of WPP for ice cream. Three ice cream mixes were formulated (Table 1) to contain equivalent total solids (37%), fat (11%), and sugar (21%), but with 3 levels of lactose (3.8%, 5.8%, and 7.8%) and added sugar (17%, 15%, and 13%). It was hypothesized that the sandy defect would become notable and ice cream with 7.8% lactose would become unacceptable to consumers within 7 mo postproduction.

**Table 1:** Base white mix formulations (% ingredient; wt/wt) before flavoring and freezing ice cream

Ingredient	Lactose mix		
	3.8%	5.8%	7.8%
Whey protein concentrate (WPC80, CROPP Cooperative, La Farge, WI)	1.3	0.6	0.0
Whey permeate powder (VersiLac, Proliant, Ankeny, IA)	0.0	1.7	3.3
Nonfat dry milk (Diamond Crystal Brands, Atlanta, GA)	2.3	3.6	4.9
Whole milk (Anderson Erickson Dairy, Des Moines, IA)	49.8	49.4	48.8
Heavy cream (Anderson Erickson Dairy, Des Moines, IA)	27.9	28.1	28.0
Sugar (Cargill, Wayzata, MN)	17.4	15.3	13.3
Stabilizer/emulsifier (Cremodan IcePro, IFF, New Century, KS)	0.5	0.5	0.5
Water (tap)	1.0	1.2	1.3

In March 2021, ice creams were manufactured in the Iowa State University (**ISU**) Creamery (Ames, IA). Weighing and blending (Rotosolver 90RS70SS, Admix Inc., Londonderry, NH) of ingredients, batch pasteurization (≥68.3°C for 30 min; C. van't Riet; Dairy Technology USA LLC, Dubois, PA), and aging (>8 h; 4°C) occurred on d 1. One 67-kg batch of the 3.8% lactose mix and one 67-kg batch of the 7.8% lactose mix were made. The 5.8% lactose ice cream base mix was made by combining 29 kg of the 3.8% mix with 29 kg of the 7.8% mix.

On d 2, vanilla flavoring (Weber Flavors, Wheeling, IL) was added before batch freezing in a Taylor Dual-Barrel (Taylor 878433B000; McCormack Distributing Company, Le Mars, IA) ice cream machine. Soft ice cream was drawn (−7°C) into 3-gallon (3.8 L) cardboard (OCTAPAK, Negus, Madison, WI) and 0.95-L containers (Belinlen, Amazon.com), then transported to a freezer (−23°C) for hardening and storage. In April 2021, 3-gallon tubs of each ice cream were transported to Kansas State University (**K-State**) at −23°C in insulated cold packs containing dry ice, then stored in a freezer (−23°C) at the K-State Dairy Processing Plant (Manhattan, KS).

This study was approved for the participation of human subjects by the ISU Institutional Review Board (IRB #20–227). Six experienced dairy products evaluators received a minimum of 4 additional hours of training, using established (Collegiate Dairy Products Evaluation Contest, 2022), and new terminology to evaluate 8 body and texture (crumbly, density, icy, gummy, melt rate, melt viscosity, greasy, and sandy), and 6 flavor attributes (cooked, lacks freshness, nonfat dry milk [**NFDM**], sweetness, vanilla, and whey) on quality characteristics of ice cream. References representing slight (0 to 5), definite (5.1 to 10), and pronounced (10.1 to 15.0) intensities of the characteristics were practiced using a 15-cm line scale. Ice creams were evaluated once or twice monthly. Trained panelists earned $5 for each training session and tasting session completed.

This study was a cooperation between ISU and K-State, and ISU served as the primary institution. The ice cream study was one component of a larger study, designed to investigate the effect of dairy nutrition educational messages on inadequate dairy consumers (Myers et al. 2024). Research participants were recruited through a Qualtrics (Provo, UT) screening survey, which was disseminated via listservs, accessed by the ISU Office of the Registrar (for students) and ISU Human Resources (for faculty and staff); the K-State Registrar approved access to all student emails, and the 2020–2021 K-State faculty senate president approved access to all K-State faculty emails. The screening surveys were launched in Iowa in January, and in Kansas in May and August. Panelists were screened based on their dairy food purchasing and consumption patterns and whether or not they had an allergy or intolerance to dairy foods. Individuals selected to participate in the study were invited to a nominal focus group (**NFG**).

Ten NFG sessions were conducted in Ames, Iowa, in April 2021; 6 NFG were conducted in Manhattan, Kansas, in July 2021; and 8 NFG were conducted in Manhattan, Kansas, in October 2021. Upon arrival, panelists were asked screening questions, handed folders with 3-digit codes, and physically distanced at sanitized tables that contained a napkin, bottle of water, sanitized pen, and cuspidor. All panelists were seated facing the facilitators, who conducted each NFG according to a script.

In preparation for NFG, ice creams were removed from frozen storage at least 24 h before the tasting event, tempered to approximately −12°C in a freezer, and scooped into 59-mL plastic sample cups with lids (Monogram, Cisco, San Jose, CA). The ice cream sample cups were maintained in a cooler with foam refrigerant ice packs (R-Freez-R-Brix, Polar Tech, Genoa, IL) in the rooms where NFG were held.

The ice cream acceptability test was conducted at a running time of approximately 32 to 37 min into each NFG. Samples were distributed one at a time to panelists, in a randomized order in April and July NFG. However, due to a change in K-State's COVID-19 protocols, October panelists received all 3 ice cream samples, simultaneously, in Styrofoam (DuPont, Wilmington, DE) trays, which maintained the temperature of the ice cream during the tasting segment. Panelists rated their liking of appearance, sweetness, flavor, texture, and overall liking on a paper ballot that contained a 5-point hedonic scale, anchored with the terms dislike very much (1), dislike slightly (2), neither dislike nor like (3), like slightly (4), and like very much (5).

After sensory evaluation, facilitators revealed the nutrition facts panels and ingredients statements for each of the ice cream samples tasted. The script included information about how the level of lactose in the 3 samples differed because of different dairy ingredients (WPC80 and WPP). It was explained that since lactose is not an added sugar, to balance carbohydrates in the mix, the ice cream with 3.8% lactose had the highest added sugar, the ice cream with a standard level of lactose (5.8%) had a moderate level of added sugar, and the ice cream with 7.8% lactose had the lowest added sugar. Panelists were given opportunities to ask questions.

After analysis of the 1-, 4-, and 7-mo data, it was discovered that ice cream quality (trained panel) and acceptability (consumer panels) did not drop significantly between mo 1 and 7. Thus, it was decided to conduct an additional consumer panel and trained evaluation at mo 10 (February). No NFG were associated with the panel, and the panel was not limited to inadequate dairy consumers. A 6-point liking scale was utilized, wherein 1 corresponded to dislike very much, 2 = dislike moderately, 3 = dislike slightly, 4 = like slightly, 5 = like moderately, and 6 = like very much, so mo-10 data were transformed to match the 5-point scale used for the April, July, and October NFG for subsequent statistical analysis. Additionally, to provide insight related to sweetness acceptability noted in the NFG, instead of sweetness acceptability, a just about right (**JAR**) analysis was conducted (options included not sweet enough [1], just about right [2], and too sweet [3]).

Untrained consumer data collected in Iowa and Kansas were entered into Excel (Microsoft Corp.), then uploaded into JMP Pro 16 (SAS Institute Inc., Cary, NC). Statistical analyses of ice cream acceptability data and trained panel attribute intensity data were performed using one-way ANOVA with Tukey post hoc tests when *P* < 0.05. Wilcoxon signed-rank tests (a = 0.05) were conducted on matched pairs to determine if NFG participants preferred any ice cream over another for any attribute.

Based upon descriptive analysis by trained panelists (n = 4 to 6), across the entire 10-mo study, the ice creams were representative of typical fresh premium vanilla ice cream with low (~50%) overrun. Quality characteristics were expected to deteriorate during storage; however, all 3 ice creams maintained low mean scores (<5.25/15.0 cm) for crumbly, icy, greasy, sandy, cooked, lacks freshness, NFDM flavor, and whey; moderate mean scores (6.5–8.5/15.0 cm) were maintained for density, gummy, melt rate, melt viscosity, sweetness, and vanilla (Table 2).

**Table 2:** Trained panelist mean scores (0.0 to 15.0 cm) for ice cream attributes with significant treatment × month effects and consumer panelist mean acceptability^1^ scores

Months of frozen storage completed	Lactose concentration (%)	Crumbly	Gummy	Sandy	Appearance acceptability	Sweetness^2^ acceptability	Flavor acceptability	Texture acceptability	Overall acceptability
1 (April; Iowa)	7.8	0.08^b^	5.22^ab^	0.08^b^	4.0^a^	4.0^a^	3.8^ab^	4.1^a^	3.9^ab^
n = 6 trained	5.8	0.08^b^	4.98^b^	0.10^b^	3.9^a^	3.8^a^	4.0^ab^	3.9^a^	4.0^ab^
n = 94 consumer	3.8	0.03^b^	5.47^b^	0.03^b^	4.0^a^	3.7^a^	3.8^ab^	3.7^a^	3.8abc
4 (July; Kansas)	7.8	0.00^b^	7.25^ab^	0.00^b^	3.7^a^	3.7^a^	3.8^ab^	3.9^a^	3.7abc
n = 4 trained	5.8	0.00^b^	7.03^a^	0.00^b^	4.2^a^	4.3^a^	4.1^ab^	4.3^a^	4.1^ab^
n = 44 consumer	3.8	0.00^b^	6.55^ab^	0.13^b^	4.1^a^	3.8^a^	3.8^ab^	3.9^a^	3.8abc
7 (October; Kansas)	7.8	0.01^b^	7.03^ab^	0.16^b^	4.0^a^	4.1^a^	3.8^ab^	3.7^a^	3.8abc
n = 4 trained	5.8	0.00^b^	6.89^ab^	0.03^b^	4.1^a^	4.0^a^	4.0^ab^	4.0^a^	3.9abc
n = 66 consumer	3.8	0.00^b^	6.95^ab^	0.01^b^	4.0^a^	3.8^a^	3.5^b^	3.8^a^	3.7^bc^
10 (February; Iowa)	7.8	3.84^a^	7.30^ab^	5.25^a^	3.9^a^	JAR 1.9^a^	3.5^b^	2.6^b^	3.3^c^
n = 4 trained	5.8	0.00^b^	8.10^a^	0.00^b^	4.1^a^	JAR 2.0^a^	4.3^a^	4.3^a^	4.3^a^
n = 55 consumer	3.8	0.13^b^	7.85^a^	0.00^b^	4.0^a^	JAR 2.1^a^	3.9^ab^	4.0^a^	4.0abc

a–c Within a column, means not sharing the same superscript significantly differ (*P* < 0.05).

1 Acceptability scores: 1 = dislike very much; 2 = dislike slightly; 3 = neither dislike nor like; 4 = like slightly; 5 = like very much.

2 Sweetness acceptability was evaluated in mo 1, 4, and 7; just about right (JAR) sweetness (1 = not sweet enough; 2 = just about right; 3 = too sweet) was rated only in mo 10.

Through mo 7, all 3 ice creams were acceptable to (liked slightly by) consumers (Table 2). However, by mo 10, consumer texture acceptability of HL ice cream dropped lower than all other texture acceptability scores; flavor acceptability and overall acceptability of HL ice cream dropped lower than SL ice cream (*P* < 0.05). Simultaneously, trained panel crumbly and sandy mean scores increased for HL, and gummy mean scores increased for RL and SL ice creams (*P* < 0.05). The negative effect of the crumbly and sandy defects is visually shown in the principal component analysis (Figure 1), wherein 63.4% of explained variance was driven by component 1. The HL ice cream evaluated in mo 10 loaded with crumbly and sandy, opposite of all acceptability vectors. Sweet and whey loaded along component 2, which explained 22.9% of the variance.

**Figure 1:**
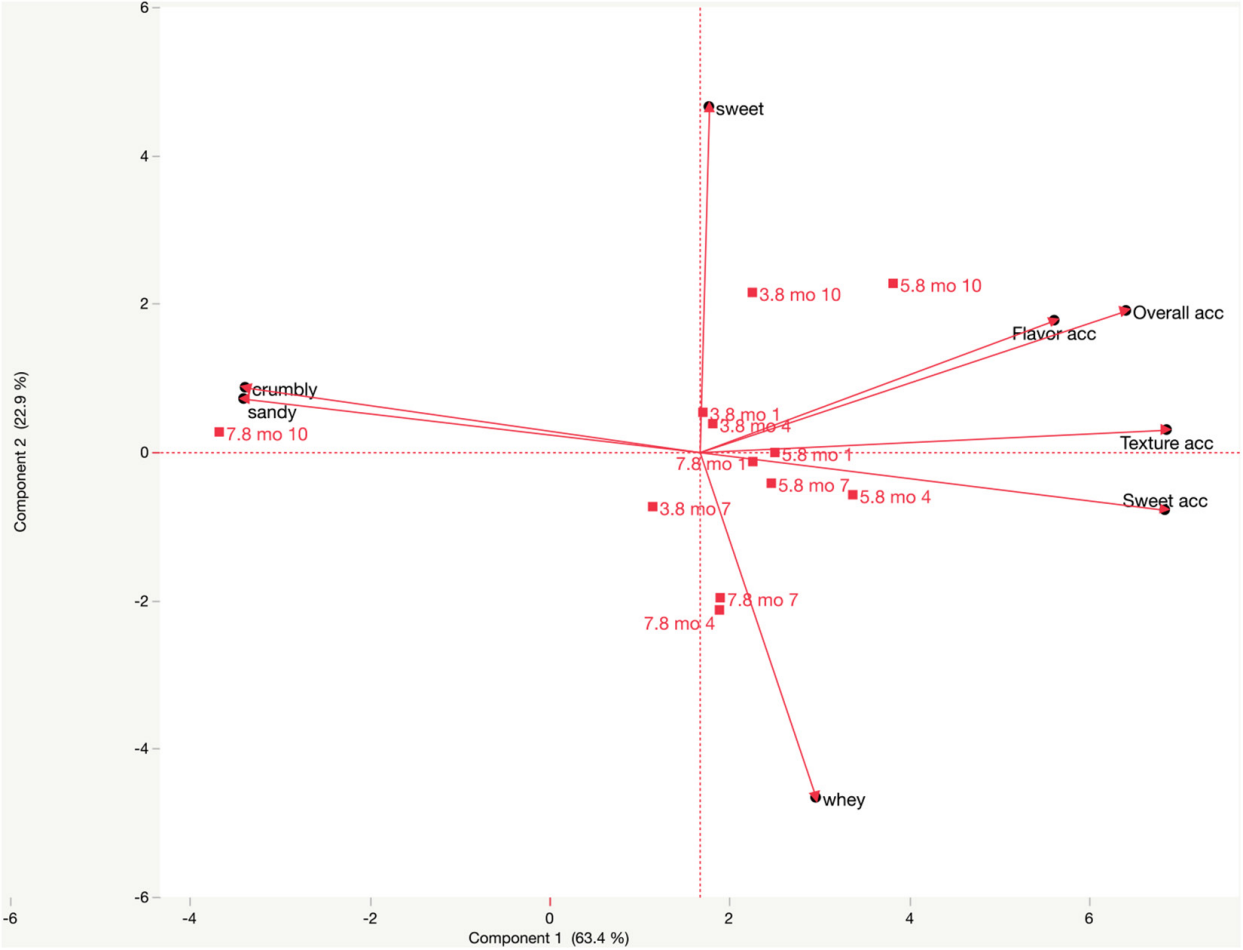
Principal component analysis biplot of meaningful descriptive attributes and consumer acceptability (acc) loadings for ice creams with reduced (3.8%), standard (5.8%), or high lactose (7.8%) after 1, 4, 7, and 10 mo of storage.

Nickerson (1962) reported that the sandy texture is promoted during typical freeze-thaw cycles, with a critical temperature between −23°C to −18°C. In the current study, temperatures fluctuated between −25°C and −15°C on a daily basis, and sandy did not show up until mo 10, suggesting potential for ice cream with WPP (SL and HL) to withstand typical retail and household freeze-thaw cycles for at least 7 mo. Temperature abuse studies are encouraged to determine whether ice cream with WPP is more, equally, or less susceptible to the sandy defect than other dairy ingredients (e.g., NFDM, WPC80).

To determine if individual panelists preferred one sample over another, differences in individual acceptability scores were tested using the Wilcoxon signed-rank test (data not shown). Panelists liked the ice cream (flavor, texture, and overall) made with the typical amount of lactose (SL) more than the RL and HL ice creams (*P* < 0.005). On the other hand, panelists preferred SL and HL sweetness over RL ice cream sweetness (*P* < 0.05). The JAR test in mo 10 helps explain the finding (Table 2); the RL ice cream was rated slightly “too sweet,” the HL ice cream was slightly “not sweet enough,” and the SL ice cream was “just about right” (*P* = 0.05). These findings align with the trained panel results (data not shown): mean sweetness of RL ice cream was higher (*P* < 0.05) than the ice creams with SL or HL. The findings are not surprising, considering that lactose is less sweet than sucrose, and the RL ice cream had the most added sucrose.

Because of its significantly lower cost, substitution of WPC80 (approximately $4.96/kg) with WPP (approximately $0.57/kg) in ice cream applications, at similar usage levels as described here, could save a producer hundreds to thousands of dollars per production run.

In conclusion, the level of lactose, in otherwise similar formulations of ice cream, did not detrimentally affect the quality or acceptability of ice cream for at least 7 mo of storage. After 10 mo of storage, ice cream with HL exhibited sandy and crumbly defects, and was less acceptable than ice cream with RL or SL. Wilcoxon signed-rank matched pair testing revealed that consumers preferred the SL ice cream flavor, texture, and overall more the RL or HL ice creams, though mean scores for acceptability were similar. Taken together, the findings demonstrate the potential for acceptable ice cream to be made with WPP, a less expensive source of dairy solids than WPC80, even at lactose levels previously considered too high for acceptable ice cream.
